# Infectious Triggers in Periodontitis and the Gut in Rheumatoid Arthritis (RA): A Complex Story About Association and Causality

**DOI:** 10.3389/fimmu.2020.01108

**Published:** 2020-06-03

**Authors:** Burkhard Möller, Florian Kollert, Anton Sculean, Peter M. Villiger

**Affiliations:** ^1^Department for Rheumatology, Immunology and Allergology, Inselspital—University Hospital of Bern, Bern, Switzerland; ^2^Department of Periodontology, School of Dental Medicine, University of Bern, Bern, Switzerland

**Keywords:** rheumatoid arthritis, periodontitis, intestinal, mucosa, trigger

## Abstract

Rheumatoid arthritis (RA) is a systemic immune mediated inflammatory disease of unknown origin, which is predominantly affecting the joints. Antibodies against citrullinated peptides are a rather specific immunological hallmark of this heterogeneous entity. Furthermore, certain sequences of the third hypervariable region of human leukocyte antigen (HLA)-DR class II major histocompatibility (MHC) molecules, the so called “shared epitope” sequences, appear to promote autoantibody positive types of RA. However, MHC-II molecule and other genetic associations with RA could not be linked to immune responses against specific citrullinated peptides, nor do genetic factors fully explain the origin of RA. Consequently, non-genetic factors must play an important role in the complex interaction of endogenous and exogenous disease factors. Tobacco smoking was the first environmental factor that was associated with onset and severity of RA. Notably, smoking is also an established risk factor for oral diseases. Furthermore, smoking is associated with extra-articular RA manifestations such as interstitial lung disease in anatomical proximity to the airway mucosa, but also with subcutaneous rheumatoid nodules. In the mouth, *Porphyromonas gingivalis* is a periodontal pathogen with unique citrullinating capacity of foreign microbial antigens as well as candidate RA autoantigens. Although the original hypothesis that this single pathogen is causative for RA remained unproven, epidemiological as well as experimental evidence linking periodontitis (PD) with RA is rapidly accumulating. Other periopathogens such as *Aggregatibacter actinomycetemcomitans* and *Prevotella intermedia* were also proposed to play a specific immunodominant role in context of RA. However, demonstration of T cell reactivity against citrullinated, MHC-II presented autoantigens from RA synovium coinciding with immunity against *Prevotella copri* (*Pc*.), a gut microbe attracted attention to another mucosal site, the intestine. *Pc*. was accumulated in the feces of clinically healthy subjects with citrulline directed immune responses and was correlated with RA onset. In conclusion, we retrieved more than one line of evidence for mucosal sites and different microbial taxa to be potentially involved in the development of RA. This review gives an overview of infectious agents and mucosal pathologies, and discusses the current evidence for causality between different exogenous or mucosal factors and systemic inflammation in RA.

## Introduction

Rheumatoid arthritis (RA) is a common immune mediated inflammatory condition primarily affecting the joints. Despite the well-described contribution of a genetic background predominantly at the immunological synapse and the many other candidate autoantigens, the origin of this potentially devastating human disease is still enigmatic. Increasing efforts have been made in the recent past to unravel the interaction of affected subjects with their environment, but many aspects of a multitude of potential triggering factors and their respective contribution in RA pathogenesis are still unknown. The mucosal surface of the oral cavity and the gut is physiologically colonized by commensal microbes, which possess the capacity to profoundly shape the repertoire of adaptive immune responses. It is one of the most fascinating current perspectives to employ this way of immune system regulation for therapeutic or preventive purposes.

An immune response against citrullinated peptides is the most specific immunological marker of RA. Citrullinated peptides are abundant in many types of inflammation, RA synovitis with all antigens for the most relevant fine-specificities of anti-citrullinated protein antibodies (ACPAs) (1–5), in extra-articular RA manifestations (6), but also in non-RA related inflammation (7) as well as in *Porphyromonas gingivalis* (*Pg*.) induced periodontitis (PD) (8). Already in the pre-clinical phase, RA patients develop ACPAs against an increasing numbers of epitopes (9). Affinity maturation of ACPA paratopes appears to cause the antigen spreading (10), but little is really understood or even proven how this phenomenon occurs. A persistent response of ACPA expressing plasmablasts predominantly of an IgA isotype suggests one or several persistent mucosal triggers in this process (11). Moreover, the highest diagnostic specificity of IgA-isotypic ACPA further supports the assumption that the most specific immune system activation in RA is happening at mucosal sites (12).

In the following chapters, we try to review parts of the overwhelming amount of data which we think is of most probable relevance. We will follow different currently proposed tracks of RA pathogenesis from genetic and environmental risk factors to microbial species and microbial communities, from innate inflammatory to adaptive immune responses and ultimately to associations with some of the characteristic features of RA. In order to sensitize the readers, we strongly recommend to scrutinize the proposed relationships in view of the Bradford–Hill criteria for causality (13). Among them, we believe that the highest attention should be given to the reported strengths of association, reproducibility, specificity, temporality, and the overall coherence of epidemiological and experimental findings.

## Inborn Factors in RA

RA is apparently not very strong clustered in families, but the genetic background of RA was in the focus of pathogenesis oriented research until the decryption of the human genome at the turn of the millennium and in the following years (14, 15). In a nationwide sibling study in the UK from 1993, monozygotic twins had about four times higher RA concordance rates than dizygotic twins (16). More recently, in the largest registry study on the inheritance of RA in Sweden, the odds for RA heritability was about three in first degree relatives and about two in second degree relatives, irrespective of the affected being parent, sibling or offspring (17). Both studies independently point to a significant genetic background of RA, which may confer to about 50% of disease risk. Today genetics in RA are still an important aspect of research with a new focus on personalized medicine, as an individually tailored approach for the minimization of the large inter-individual variability response to therapy (18).

The strongest genetic risk factor for RA is a specific peptide sequence in the type II human leukocyte antigen (HLA) or major histocompatibility complex (MHCII) of only six amino acids, called the shared epitope motif. Shared epitope motifs are especially frequent in native Americans (19). MHCII molecules play a crucial role in the presentation of antigens, and their association with RA is a consistent finding in many populations of different ancestry (20–24). MHCII molecules are central in directing adaptive immune responses. Only a few alleles in the DRB1 molecule, which are coding for QKRAA [Q (glutamine) K (lysine) R (arginine) and AA (alanine-alanine], QRRAA, or RRRAA amino acid sequences in the positions 70–74 of the third hypervariable region, have a strong association with RA. However, other alleles in the HLA complex (25) as well as dozens other non-HLA genes appear to also confer to the genetic risk of RA, but to a much lesser extent (26). Other sufficiently robust RA associated genes are single nucleotide polymorphism (SNP) in the PTPN22 gene, which codes for a non-receptor lymphoid protein phosphatase and negative regulator of presentation of immune complex derived antigens (27) and a specific allele in human PADI4 (28). Other genetic associations were too inconsistently associated with RA to mention in this brief overview.

Recently, X-ray crystallography studies could demonstrate citrullinated as well as non-citrullinated vimentin peptides in the binding groove of HLA-DRB1 molecules (29). This finding suggests that the genetic background of MHC molecules is directly linked to the antigenicity of specific peptides. Interestingly, recent data demonstrate an effect of the shared epitope on the gut microbiome in clinically healthy study populations (30). Thus, it is tempting to speculate that RA-related MHC alleles affect the presentation of disease relevant antigens and the symbiotic coexistence of the host and its microbiota by the same key MHCII molecules. However, other researchers suggested an alternative and probably antigen independent explanation for the association of MHC molecules with RA (31, 32).

Another inborn X-chromosomal risk factor for RA is the female sex. Notably, although female subjects are about two to three times more often affected by RA in the general population, familial RA aggregation appears not to be affected by sex (17). Female RA preponderance seems to be limited to the reproductive phase of life, but late onset RA appears to be similar prevalent in male and in female (33). We currently have no consistent data on an inappropriate inactivation of X-chromosomal genes in RA (34). Furthermore, as indicated by the preferential disease onset in the menopause, RA onset or flares in the first year after delivery but treatment-independent amelioration of disease activity during pregnancy, the role of the female sex hormones in RA appears to be complex (35, 36). As for all large epidemiological studies, it has to be kept in mind that the results are strongly depending on the robustness of disease definition, e.g., autoantibody status, which can be a major challenge in the field (17). Furthermore, the strength of observed association with sex appears to be affected by ancestry, by disease severity, by disease onset during life time or parity (37). Although the research field on the vaginal microbiome and female health is rapidly growing, we did not retrieve any specific literature on this topic in relation to RA.

## Age and Behavioral Risk Factors for RA

RA as well as RF and ACPA associated types of juvenile idiopathic arthritis may start at any phase of lifetime. However, RA incidence is highest in the fifth and sixth life decade. This fact may hint to the important non-genetic factors, which become only active under certain circumstances. Life style factors also appear to be relevant, as age- and sex-standardized incidences were lower in densely populated areas and in individuals with high educational level (34). Depending on sex, RA occurred in a study from Sweden more often in male farmers, brick layers, and electric or electronic workers, and in female preferentially in nurse assistants and social science related workers (38).

Smoking is one of the best established environmental risk factors especially for RF-positive RA and especially in men (39). Tobacco smoking was the first environmental factor that was associated with the onset RA (40, 41), but smoking can explain the severity of RA only to some extent (42). Furthermore, smoking is associated with extra-articular RA disease manifestations such as interstitial lung disease (43) and subcutaneous rheumatoid nodules (44). The mechanisms of how smoking might affect RA must be further elucidated. Moreover, with the given focus of this review, smoking is also an established risk factor for periodontitis (45).

The influence of diet on the onset and course of RA is since a long time a matter of an intensive debate. Mediterranean diet as well as antioxidant and fruit-rich diet have been proposed to be protective (46–48), while obesity seems to have negative effects on the risk for the development of RA. However, any observed effects of diet on the RA disease risk were rather small, and even bariatric surgery appeared to be without effect on RA status, despite its obvious consequences for the nutritional status as well as for the intestinal microbiome (49, 50). Coffee or tea consumption appears to be irrelevant for the onset of RA (46). Alcohol consumption in contrast to smoking does not seem play a relevant role in the incidence of RA (51), but appears to have modest effects on PD (52).

## Invasive Infectious Triggers

One of the most frequent causes of an inflammation is an invasive infection. However, RA in contrast to reactive arthritis starts very rarely with a clinically apparent infection. Following the classic postulates of Robert Koch for the proof of a microbial origin of disease, RA would not be proven infectious origin (53). However, an imperfect but repeatedly significant association of specific MHCII alleles necessary to develop RA may indicate a relevant role of host response mechanisms for an infection with low disease penetrance, which could have prevented the discovery of an infectious origin of RA.

Following the first of Koch's postulates of an infectious disease origin, a microbial agent or at least some of its components should have been detected in RA joints. A landmark study on this topic was published in 2003 (54), when authors searched for bacteria-derived muramic acid by gas chromatography-mass spectrometry (GC-MS) as well as bacterial 16s or 23s rRNA by polymerase chain reaction in RA synovium. This study was positive in a few patients with longstanding RA, but in similar frequency as in control subjects (54). In another study, bacterial DNA from *Pg*. was identified in 15% of RA samples, which was significantly more frequent than in the 3% of synovial fluid from control subjects (55).

Zhao et al. (56) reported the presence of bacterial 16s rRNA from many different species in synovial materials from RA and control samples, which draws any species-specific invasive infection to cause RA into question. However, this notable finding should be confirmed in an independent study. An invasive infection in RA must not necessarily be proven in the joint. In RA associated vasculopathy, *Methylobacterium oryzae* was detected in the aortic adventitia in 3 out of 11 biopsies, but different bacterial species were detected by 16s rRNA sequencing in 4 out of 11 control samples (57). As far as we know, *Methylobacterium oryzae* has never been isolated from RA joints.

Viral infections are since a long time handled as a potential infectious trigger of RA. In a recent systematic review, an overall poor quality of studies on RA incidence upon viral exposure was reported. The risk of RA onset appeared to be somewhat increased after Parvo B19 [*n* = 12 studies, OR = 1.77 (95% CI: 1.11–2.80), *p* = 0.02], hepatitis C virus [*n* = 7 studies, OR = 2.82 (95% CI: 1.35–5.90), *p* = 0.006] and possibly also after EBV infection (58). In summary, we have some evidence for infectious triggers, but only limited evidence for an invasive infection causing RA. Furthermore, all the few positive studies for an invasive infection in RA are still awaiting independent confirmation.

## Disease Models for Mucosal Infections and Dysbiosis

In animal models, major effects of oral as well as of intestinal infectious triggers could be observed on incidence and severity of arthritis (Table 1). Furthermore, inoculation of some specific periopathogens in the oral cavity appeared to affect the composition of the gut microbiome. However, there also exists experimental evidence from the K/BXN serum transfer model in C57BL/6 mice that an existing arthritis might not only be consequence of intestinal dysbiosis, but may act back on mucosal inflammation by down-regulation of several pro-resolving mediators (59). As compared to control mice, the gut protective mediator resolvin became metabolized to its inactive 17-oxo metabolite, when arthritis was induced by K/BxN serum transfer. Furthermore, the mucosal expression of anti-inflammatory IL-10, the number of goblet cells and the expression of tight junction molecules was reduced in arthritic mice, thereby increasing the gut permeability for microbes (59). Increased gut mucosal permeability upon serum transfer was further aggravated upon *Pg*. inoculation directly in the stomach, but administration of resolvin in arthritic *Pg*.–inoculated mice normalized mucosal IL-10 expression and gut permeability and ameliorated arthritis. This study elegantly demonstrates how a weakened gut barrier can be critical for the pathogenic action of intestinal microbes (59).

**Table 1:** Mucosal inflammation in arthritis models.

**Model**	**Animals**	**Challenge**	**Microbial stimulation**	**ACPA status**	**References**
K/BxN	C57BL/6	K/BxN serum	Intestinal *Pg*. on three occasions	Not reported	(59)
CIA	BALB/c	CII + FA	*Pg*. after 3d antibiotics	Unknown	(60)
AIA	DR4-IE-tg	FA	CEP-1 and REP-1 from human and *Pg*.	Positive	(61)
	MHC II (–/) wt				
	C57BL/6				
CIA	DBA/1	CII + FA	*Pg*. W83 wt. and PPAD-	Positive	(62)
CIA	DBA/1	CII + FA	Oral *Pg*. infection	Unknown	(63)
CIA	BALB/C	CII + FA	*Pg*. vs. PPAD def. *Pg*.	Positive	(64)
CIA	B10.RIII mice	CII + FA	*Pg., T. denticola, T. forsythia*	Unknown	(65)
CIA	(HLA)-DR1 humanized C57BL/6	CII + FA	Oral *Pg*. infection after 7d SMZ-TMP	Positive	(66)
SKG	ZAP-70 mut	Laminarin	*Pg*. i.p.	Positive	(67)
CIA	DQB1-tg B6	CII + FA	*Prevotella histicola*	unknown	(68)
CIA	DBA/1	CII + FA	*P. gingivalis, P. intermedia*	Pos., unchanged	(69)
CIA	DBA/1	CII + FA	Antibiotics	Unknown	(70)
AIA, CIA	TH17–/– C57BL/6	CII+ FA	Antibiotics, Jackson microbiota	Unknown	(71)
CIA	F1 (DBA/1 × B10.Q)	CII + FA	*P. gingivalis*	Unknown	(72)
CIA	DBA/1	CII + FA	Antibiotics before and after challenge	Negative	(70)
	Lewis rats		*P. gingivalis, P. intermedia*	Positive	(73)

*CII + FA, Type II collagen and FA Freund's adjuvant; CEP, citrullinated alpha enolase from human or Pg.; K/BXN serum transfer model, Serum from F1 generation of T-cell receptor transgenic KRN mice with autoimmune-prone non-obese diabetic (NOD) mice (74). Pg., Porphyromonas gingivalis; REP arginine bearing alpha enolase from human or Pg*.

In the study of Flak et al. (59), an oral pathobiont was directly inoculated into the stomach, thereby preventing Pg-induced PD. In the meantime, a correlation of oral and intestinal mucosal colonization was confirmed for several times and in different arthritis models (Table 1). However, most of the data come from collagen induced arthritis (CIA), which typically develops a rapidly erosive but self-limiting disease without citrulline specific immune responses. Nevertheless, as a conclusion of a rapidly increasing number of animal experiments, we have strong evidence that arthritis-relevant triggers of the immune system could be initiated by commensal or facultative human pathogens in the oral as well as in the intestinal mucosa.

## Periodontitis

Healthy squamous epithelium of the mouth or cylinder epithelium of the gut and respiratory mucosa should represent a sufficient defense line against invading microbes of low virulence. However, these mucosal tissues show important anatomical differences, which may warrant more attention than what is currently reported.

The gingival mucosa especially in the close proximity of teeth represents a weak point in the barrier against invasive microorganisms. The periodontal tissue is perfect site for longstanding commensal colonization and a nidus of dysbiotic biofilm for a permanent immune stimulation. With a 3.47 billion people estimate (95% CI: 3.27–3.68), oral disorders are globally the number one among all level three burden of disease conditions (75). Nutritional components such as carbohydrate intake and other behavioral factors such as standard and habits of dental hygiene as well a smoking are likely to have a major influence on the microbial colonization and thus on both the evolution of caries and periodontitis during lifetime. According to recent global estimates, 743 million people worldwide are affected by severe PD (76).

PD is characterized clinically by bleeding or suppuration upon probing due to pocket formation and loss of supporting alveolar bone (77). In contrast, the gingivitis is characterized by a bleeding of the gingiva without pocket formation and bone loss. PD is triggered by so-called lead bacteria, which are mostly facultative anaerobic pathogens. It is assumed that these ubiquitous taxa are present in every human's oral cavity, but in such small numbers that they can be kept in check by the natural microbiota and the host immune system. Socransky categorized PD triggering bacteria into four different complexes (78), the early colonizer which are mainly streptococci, followed by so called bridge species such as Fusobacteria and *Prevotellaceae*, which create an ideal livelihood for the most aggressive microbes.

**Figure 1:**
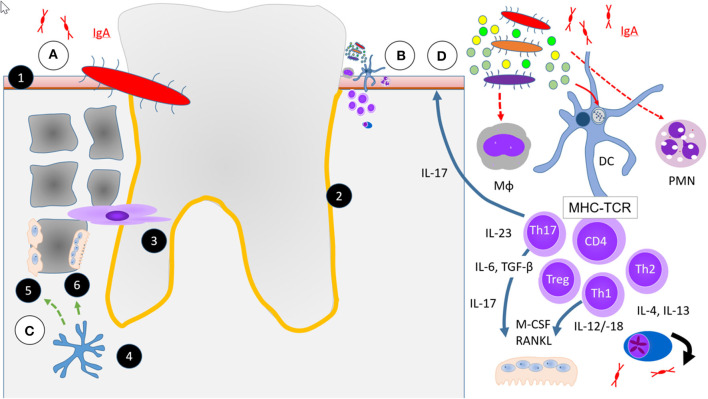
Simplified scheme of four hypotheses or aspects in the etiopathogenesis of periodontitis (PD) relevant to RA. **(A)** Falsified scenario of a single specific infectious microbial pathogen, i.e., *P. gingivalis*. **(B)** Current hypothesis of an increasing multitude of microbes necessary to initiate and maintain PD the polymicrobial synergy and dysbiosis model (PSD) and immune response in RA. **(C)** Osteoimmunology. **(D)** Excessive or unresolved inflammatory response. (1) Squamous epithelium, (2) Periodontal space (orange line), (3) Fibroblast periodontal ligament cells, (4) Osteocyte control Wnt-signaling by Sclerostin and DKK-1 (green dashed arrow) in (5) Osteoblasts and activate (6) Osteoclasts by RANKL (green solid arrow). Abbreviations for different immune cells: Mϕ are macrophages, DC dendritic cells, PMN neutrophils, CD4^+^, Th1, Th2, Th17, and Treg are some of a much larger multitude of T cell subsets in PD. MHC, major histocompatibility complex and TCR, T cell receptor represent the immunological synapse of antigen-specific immune response. All other abbreviations of soluble factors are explained in the text.

More recently, a new periodontal disease model, i.e., the polymicrobial synergy and dysbiosis (PSD) model, has been proposed (79) (Figure 1B). Peridontitis in the PSD model is initiated by a synergistic and dysbiotic microbial community rather than by select “periopathogens,” such as the “red complex.” In this polymicrobial synergy, different members or specific gene combinations within the community fulfill distinct roles which may act synergistically in order to form and stabilize a disease-provoking microbiota. In this model certain microbial species, termed “keystone pathogens” play a crucial role to modulate the host response in ways that impair immune surveillance and shift the balance from homeostasis to dysbiosis. The so called “keystone pathogens” also increase the virulence of the entire microbial community through interactive communication with accessory pathogens.

To the “key stone” pathogens belong the facultative anaerobic bacteria *Pg., Treponema denticola, Tannerella forsythia*, and *Aggregatibacter actinomycetemcomitans* (*Aa*.). These pathogens are strongly related to the flora found in deep periodontal pockets associated with advanced periodontal disease (80). *Pg*. possesses some virulence factors of special interest in the context of RA: it has its own citrullinating enzyme, Porphyromonas peptidylarginine deiminase (PPAD), which is expressed on the outer membrane of *Pg*. and differs from human PAD's in its Ca^2+^ independent enzymatic activity (62). Furthermore, PPAD in contrast to human PAD is capable of citrullinating C-terminal arginine residues, which are generated by another *Pg*.-derived enzyme, arginine specific gingipain (Rgp) protease (81). The coordinated activity of these two microbial enzymes is unique in having the capacity of generating known RA autoantigens such as C-terminal citrullinated fibrinogen and enolase without aid of human enzymes (82).

One of the first cross-sectional RA association studies with PD goes back to 1997 (83), when the nowadays available modern biological and targeted immunosuppressive therapies were not available. Many patients were at that time in advanced stages of RA and handicaps in accurately performing oral hygiene measures were likely present in this population with longstanding RA. By using nationwide health care data for PD, the number of reimbursed dental treatment courses for PD as well as the costs for PD therapy before the onset of RA were significantly increased in a large Taiwanese case-control study (84). As compared to health care insurance patients in a database without PD, patients with PD but without dental scaling (HR = 1.89, 95% CI: 1.56–2.29) had the highest RA risk, followed by PD patients who had received PD therapy (HR: 1.35, 1.09–1.67 (85). In some studies, the oral microbiome appears to be altered in RA anyway and irrespective of the co-existence of PD (86, 87), and even in orally healthy subjects (88), but the composition of the oral microbiome was not in all association studies associated with RA (89).

In RA association studies for specific periodontal microbes, *Pg*. was on basis of its citrullinating properties of self- and foreign-antigens the first candidate periodontopathogen to be studied in context of RA (90–92). Infection of the gums by *Pg*. and PD is probably not the same, as *Pg*. alone at very low colonization levels was not sufficient to cause periodontitis in germfree mice, but disrupted the host-microbial homeostasis and caused severe PD when added to a community of commensal microbiota (93, 94). Furthermore, *Pg*. in contrast to typically health associated oral commensals was eliminated from the feces, elicited systemic immune responses and induced pathological changes in the liver, which supports the importance of an oral-gut connection (93).

Better understandable in respect of these experimental findings was that the frequency of PD as well as of immune responses against *Pg*. was increased in a British study in ACPA positive subjects without arthritis (95). In a Western Chinese study, *Pg*. was expanded in patients with established RA, but reduced in ACPA positive high-risk individuals (96). ACPA positivity in contrast was not linked to immunity against *Pg*. in the French early RA study cohort (97). Furthermore, in treatment naïve patients with arthralgia, inflammation was rather linked to the presence of PD than the presence of *Pg*. (98). Moreover, PAD expression and citrullination in the periodontium was neither associated with the presence of *Pg*. nor with *Aa*., another interesting common pathogen PD in context of RA (99). In a study in patients with established RA, antibodies against *Pg*. derived arginine gingipain type B (RgpB) were associated with RA, but smoking interacted with PD as well as with RA (100). Finally, as a summary from three studies in patients with established RA, the presence of *Pg*. in the gingival crevicular fluid as well as *Pg*. directed antibody response appeared to be more closely associated with PD than with RA (86, 91, 101). Other cell- and surface receptor-specific data will be discussed in the respective chapters.

The second already mentioned periodontal pathogen with specific features of interest in context of RA, *Aa*., induces the pore-forming toxin leukotoxin-A (LtxA), thereby releasing citrullinating enzymes from neutrophils (102). A third periodontal taxa to be briefly discussed in context of RA is *Prevotella intermedia* (*Pi*.) and Prevotella_6 (P_6). *Pi*. causes citrullination of different peptides in the crevicular fluid, among them peptides from Tenascin-5. Anti-tenascin-5 antibodies were detected in 18% of pre-RA and in about 50% of sera from patients with manifest RA with a specificity of 98% (103). P_6 was identified in the Western Chinese study population in high-risk individuals for RA and in patients with established RA (96).

### Pathology of PD

Gingivitis and PD are a continuum of diseases of the teeth supporting tissues (Figures 1A–D). In 1976, Roy Page and Hubert Schroeder described PD as the host response to a lasting accumulation of dental plaque. They described the entire process in four phases, an “initial,” an “early,” an “established,” and an “advanced” stage of lesions (104, 105). Initial lesions were characterized by an inflammatory infiltrate of mainly neutrophils, early lesions predominated by macrophages and lymphocytes and later phases of PD with more complex cellular infiltrates.

In gingivitis and the initial stage of PD, the junctional epithelium starts to produce prostaglandin E2 (PGE2) and other chemotactic mediators (106). This leads to enhanced permeability of the endothelium, accumulation of numerous neutrophils, and evasion from the junctional epithelium into the gingival sulcus. The mucosal epithelium starts to proliferate and an apical migration of the junctional epithelium may be observed. Detachment of the junctional mucosa from the enamel defines disease progression from initial to an early disease stage, while deeper pockets are characteristic for an established stage. From this stage onwards the epithelial defense line is growing and the retention of the dysbiotic biofilm at the same time facilitated.

In parallel to the epithelial alterations and cellular infiltrates, the subepithelial matrix stroma becomes increasingly affected. Periodontal ligament cells and other fibroblasts start to proliferate. Furthermore, osteoblasts and other progenitor cells differentiate into osteoclasts and start to degrade the bone matrix, which is the main criterion of advanced PD. Page and Schroeder already reported from longitudinal observations that established lesions did not necessarily progress to bone resorption and edentulism, but could remain stable indefinitely. This led them to conclude that an appropriate level of host response and maintenance of a stable balance despite the persistence of a dysbiotic biofilm could be achieved in chronic PD (104, 107). Variants in the MHC II complex definitely have an important impact on the strength of adaptive immune responses, and an overwhelming amount of data from around the globe shows their importance for the development of RA (20–24). In comparison, the strongest known genetic associations for PD are observed with genes outside the MHC II complex (23, 24, 108), and they are much weaker. Thus, RA and PD are not linked to each other by their genetic background. In the following, we will discuss the potential role of different cell types for the onset of PD with a perspective of its relevance for RA.

Since the early descriptive studies of PD (107), major advances in knowledge about the biology of PD could be achieved by interventional studies in various PD models. Experiments were performed in knock-in and knock-out animals and even germfree mice. Some came to fascinating novel results that changed the view on PD, but most of these data are yet unrelated to systemic (auto-)immunity or arthritis. At this place, we like to refer to the excellent review of Hajishengallis and Korostoff (104) for a comprehensive overview, but we want to briefly discuss here some of the central results and the findings with highest probability to be relevant for RA.

### Resident Periodontal Cells

The alveolar bone is the indispensable mechanically stable ground for the fixation of the teeth and covered by the periodontal and gingival epithelium and subepithelial stroma. In between of teeth and the bone, the architecture of the periodontal tissue is defined by collagen fibers, glycoproteins, and many other macromolecules. To allow a stable fixation of the teeth in the cavities of alveolar bone, oblique, and horizontal type-1 collagen fibers are circumferentially strained around the teeth. This extracellular matrix is synthesized and continuously reshaped for the changing demands over time by resident periodontal ligament (PDL) cells and fibroblasts. However, in their capacity of producing high amounts of tissue degrading matrix metalloproteinases (MMPs) in an activated state, they resemble to some extent fibroblast-like cells in the RA synovium. PDL are also capable of phagocytosing and processing pathogenic periodontal microbes such as *Aa*. (109). Upon stimulation, e.g., with viable *Pg*., PDL express increased amounts of the inflammatory cytokines IL-1β, IL-6, TNF, as well as chemokines such as IL-8, CCL3, CXCL12, and monocyte chemotactic protein MCP-1 (110). Notably, PDL and fibroblasts of other subgingival localization appear to respond differently to inflammatory stimuli (111). However, despite their capacity of transiently elevating the expression of MHC II molecules upon stimulation with pro-inflammatory cytokines, even potently cytokine-stimulated PDL do not express CD40 or CD80 co-stimulatory molecules, which are fundamentally important characteristics of professional APC (109).

Alveolar bone loss is hardly reversible and a hallmark of the most advanced stage of PD (Figure 1C). Until recently, when we compare PD with a cacophonic symphony of the oral and dental health, microbial invasion was believed to be the conductor and the host's inflammatory response the orchestra of PD. The bone tissue was until recently exclusively believed to be the passively suffering audience, with the osteoclasts among them at best as applauding listeners. Recently, it became clear that osteocytes play an active and central role in PD by expressing receptor activator of nuclear factor kappaB ligand (RANKL) (112). Together with macrophage colony stimulating factor (M-CSF) which stimulates the proliferation of osteoclast progenitors (113), RANKL is the key stimulus of osteoclast formation (114, 115). When a mixture of *Pg*. and *Fusobacterium nucleatum*, two frequent dysbiotic bacterial strains were several times inoculated into the periodontal tissue of osteocyte-specific RANKL-deleted mice, osteoclast numbers were not increased nor were the bony surfaces eroded. In contrast, the same PD-associated bacteria mixture increased osteoclast numbers and caused severe alveolar bone loss in wild type mice (112).

Osteocytes do not only express RANKL, but they also express other mediators with major relevance for anabolic processes in the bone. Wnt/β-catenin (named by homologies to the wingless gene in Drosophila and int-1 oncogene in mice) is a major signaling pathway for osteoblast formation and differentiation (116). This pathway is of central importance in the embryonic osteogenesis and later in life for bone homeostasis. Wnt/β-catenin signaling in osteoblasts is under control of sclerostin and dickkopf-1 related protein (DKK1). Sclerostin antagonizes canonical Wnt-signaling directly by binding to the LRP5/6 receptor, while DKK1 exerts its action by binding to the Wnt co-receptor (116, 117). Osteocytes express and secrete sclerostin and DKK1 (118).

Sclerostin appears to be involved in the etiopathogenesis of PD, as knock out mice had a slightly ameliorated PD phenotype (119) and antibodies against sclerostin inhibited the progression of PD (116). Even more interesting, sclerostin antibodies were able to ameliorate inflammation and to partially revert PD related bone damage (118). In conclusion, osteocytes are a source of important mediators of bone in periodontitis. In human PD, sclerostin concentrations were locally elevated in the gingival crevicular fluid only from diseased sites (120), hereby indicating a locally restricted response of osteocytes. This finding seems to be specific for sclerostin, as concentrations of TNF and a soluble activator of the Wnt-pathway, Wnt-5a, appeared to be altered in a similar way (120). In opposite to this local finding, sclerostin in contrast to DKK1 concentrations were elevated on a systemic level in the sera of PD patients (121). In difference to PD, elevated DKK1 but not sclerostin serum concentrations were related to joint damage progression in RA (106). Furthermore, although the biological importance of sclerostin for the negative effects on bone formation were recently shown in arthritic rats (122), the periodontal bone loss in PD is restricted to the gums, and a direct link to the joint erosions in RA remains currently unexplained by soluble factors. However, it could be interesting to study the relevance of sclerostin and DKK1 on a systemic level for other bone-specific aspects of human RA such as osteoporosis, abnormal bone geometry and accelerated thinning of metacarpal bones (123–125).

### Innate Immunity

With their main function of phagocytosis and elimination of pathogens, polymorphic nuclear cells (PMN) are the dominant cell population in gingivitis (Figure 1D). Given the relevance of infectious noxes in PD, it appears rational to assume that an impaired elimination of dysbiotic bacteria by defective PMN might be critical. However, a normal frequency of PD in patients with a severe X-linked defect in the nicotinamide adenine dinucleotide phosphate (NADPH)-oxidase necessary for the respiratory burst of phagocytes (104, 126) suggests only a secondary role of bacterial elimination by neutrophils for the prevention of PD.

We have increasing evidence that a periodontopathogen such as *Pg*. may circumvent elimination despite an originally intact neutrophil biology. PMN migrate along chemokine gradients such as C′5a or C′3a complement factor concentrations through the capillary endothelium, the submucosal stroma and gingival epithelium. Intriguingly, therapeutic blockade of the C′5 receptor in a prophylactic protocol prevented PD, respectively application in a therapeutic manner alleviated PD in a *Pg*. induced periodontitis model (127). Notably, *Pg*. can use the C′5a receptor in crosstalk with toll-like receptor 2 (TLR-2) to induce proteasomal degradation of the Toll-like receptor-2 adaptor myeloid differentiation primary response protein-88 (MyD88) in neutrophils and other phagocytes (128). The resulting lack in MyD88 impairs the rapid activation of the inflammasome complex and affects the host defense against dysbiotic microbes, but the same initial process activates a phospho-inositol-3 kinase (Pi3K) dependent pro-inflammatory pathway.

TLR-2 is a pivotal receptor for innate immune processes in neutrophils, in monocytes and in macrophages (129). Activation of TLR-2 appears to be crucial for the development of PD, as TLR-2 deficient mice are normally resistant to *Pg*. induced PD (130). Adoptive cell transfer of TLR-2 positive monocytes and macrophages enables *Pg*. to induce PD in TLR-2 deficient mice (130). Furthermore, TLR-2 may mediate longer bacterial persistence in macrophages and stimulate TNF-dependent osteoclast activation (130, 131).

Macrophages are directed *in vitro* to M1 in the presence of lipopolysaccharides (LPS), granulocyte-monocyte colony stimulating factor (GM-CSF), and interferon-gamma (IFN-γ), and are characterized by CD86 surface expression, inducible nitric oxide synthase (iNOS), TNF, interleukin 1 beta (IL-1β), IL-6, IL12, and IL-23 expression. M2 macrophages in contrast origin from alternative activation in a Th2 dominated cytokine milieu with excess of IL-4 and IL-13 from Th2 differentiated T cells. M2 cells are characterized by CD206, IL-10, and transforming-growth factor beta (TGF-β) expression (132). Both, M1 as well as M2 macrophages are present in human PD, but periodontal macrophages appear to be predominantly polarized to the classically activated M1 phenotype (133, 134).

### Switching From Innate to Adaptive Immunity

A low number of lymphocytes, predominantly CD4^+^ and CD8^+^ and a few γδ T cells can be found in the healthy periodontium (Figure 1D) (135). Upon activation by antigen-presenting cells, naive CD4^+^ T cells can be polarized into distinct effector T helper (Th) cell subsets; Th1, Th2, Th17, and regulatory T (Treg) cells, depending on the local cytokine milieu.

Dendritic cells (DCs) are the best studied APCs in mucosal tissues. DCs can be subdivided into predominantly resident DCs and those with migratory potential. For the spreading of RA relevant antigens, the latter appear to be of greater interest (136). With regard to their migratory capacity, *Pg*. is capable of inducing CCR6 expression in CD1c^+^ DCs, as the CCL20 ligand of CCR6 was elevated in *Pg*. induced PD (137). Non-canonical DC maturation by *Pg*. is reported to occur with or without GM-CSF/IL-4, and *Pg*.-infected DCs become resistant to apoptosis and inflammatory pyroptosis (138).

The 67 kDa minor fimbriae Mfa-1 bacterial adhesion molecule is known for inducing the expression of dendritic cell-specific intercellular adhesion molecule-3-grabbing non-integrin (DC-SIGN) or CD209 (138). Mfa-1 is a DC-SIGN ligand, and the 41 kDa major fimbriae protein FimA a TLR2 agonist (139). DC-SIGN and TLR-2 are two different pattern recognition receptors (PRRs) on DCs, and their activation has divergent consequences for the survival of *Pg*. (138). TLR2/4 deficiency ameliorates the course of PD to the costs of more extensive bacterial spreading throughout the body due to insufficient bacterial containment or killing (140). Uptake of *Pg*. into DC by interaction of Mfa-1 with DC-SIGN resulted in lower intracellular killing and higher intracellular content of *Pg*. in single membrane phagosomes, where the bacteria survived intracellularly after prevention of phagolysosome formation. Furthermore, interaction of Mfa-1 with DC-SIGN in stably transfected monocytic cell lines induced lower expression levels of CD80, CD83, and CD86 co-stimulatory molecules, and secreted significantly lower levels of inflammatory cytokines IL-1β, IL-6, IL-8, IL-12 p70, and TNF (139). In contrast, uptake of *Pg*. in the absence of DC-SIGN upon single activation of TLR-2 and autophagy was associated with endosomal lysis and reduced survival of *Pg*. (138).

Another receptor on DCs for fimbriae proteins is C-X-C chemokine receptor type 4 (CXCR4), which is also present on DCs in RA synovium (141). Activation of the CXCR4 receptor appears to be beneficial for the severity of PD by disrupting immunosurveillance, but with the consequence of prolonged bacterial persistence (142, 143). As another interesting finding, CXCR4 inhibition appears to be beneficial in terms of a lower expression of oncogenes (144). Upon activation of the MAP kinase pathway, *Pg*. may induce protective genes against oxidative stress and apoptosis in mDCs via forkhead box class-O protein FOXO1 (143). Control of FOXO1 in DCs reduced the cleavage of caspase-3 and decreased the expression of pro-apoptotic proteins Bax and Bim (144). Myeloid DCs had a better longevity and propagated the generation of local Treg by Indole amine 2,3 dioxygenase (Ido1) activity in the presence of *Pg*. (144).

FOXO1 transcription factors appear to be essential for the mucosal immunity, as they do not only regulate DCs, but pro-inflammatory signaling molecules (TLR-2, TLR-4, IL-1β, and TNF), wound healing factors such as TGF-β and vascular endothelial growth factor (VEGF), integrins, a proliferation inducing ligand (APRIL) and B-lymphocyte stimulator (Blys), T-regulatory modulators (Foxp3 and CTLA-4), antioxidants and DNA repair enzymes in different immune relevant cell populations (145). In conclusion, *Pg*. infected and apoptosis resistant mDC can lead to local immunological tolerance, but are at the same time a good candidate for spreading the key pathobiont of PD into other organs and tissues.

CD207^+^ (langerin) positive Langerhans cells (LC) are potent immune regulators, but are in contrast to conventional myeloid DCs resident cells predominantly in the periodontal epithelium (146). They are important mediators of *Pg*. induced local Th17 differentiation, but have only little effect on the migratory capacity of conventional mDCs (146). Myeloid CD207-DCs could migrate from the lamina propria into the regional lymph nodes. We speculate that non-Langerhans DC are more likely to have an impact on systemic immune response or microbial spreading. Furthermore, the generation of Th1 cells as well as regulatory T cells was not affected in mice lacking LC (146). Notably, despite a deficiency of Th17 cells, alveolar bone resorption by osteoclasts was not affected by a lack of LC (146).

### Adaptive Immune Response

T and B cell infiltrates are abundantly present in established and in advanced PD (104) (Figure 1D). T cells become locally primed, according to the dominating cytokine milieu, into Th1, Th2, or Th17 cells, to provide locally active inflammatory, regulatory, or immune activating signals. Th17 cells are divided into two subsets; homeostatic Th17 cells which accumulate in the periodontal space in an IL-6 dependent manner, and locally expanding Th17 cells which require both, IL6 and mostly monocyte derived IL-23 for local expansion. Genetic as well as therapeutic blockade of IL-17 diminished the amount of inflammatory response in PD as well as bone loss, but propagated fungal infections (147).

Plasma cells are also present in chronic PD. The occurrence of IL-35 and IL-37 expression appears to be beneficial to the local inflammatory process, as both cytokines inhibited osteoclast formation at least *in vitro* (148). However, these findings appear to be controversial to other studies, and we retrieved surprisingly little original data on the role of plasma cells as local antibody secreting cells (149).

Probably more interesting for the systemic aspects of mucosa driven autoimmunity in RA is the total lack of reports regarding the formation of lymph follicles or other organized lymphoid structures, which appears notable in view of more than 500 histological studies that were performed in relation to PD. Thus, any canonical immune response in relation to mucosal infections requires the migration from the affected tissue to the regional lymph nodes.

### Effects of PD Treatment on RA

More intensive than only standard hygienic means are necessary in the advanced stages of PD, when deeper pockets prevent an efficient reduction of pathogenic bacteria from heavily colonized dental plaques (76). Standard of care in advanced PD is non-surgical scaling and root planning (SRP) plus intensive oral hygiene, e.g., with antimicrobial chlorhexidine containing mouth rinse. This procedure reduces the periodontal microbiota at least transiently is called a one-stage full mouth disinfection (FMD), which may be completed by short term systemic antibiotic therapy (150–152). In a recently published randomized controlled trial in patients with PD plus RA, no significant effect on RA disease activity was demonstrated upon standard PD therapy (153). However, although the autoantibody profile against citrullinated peptides remained unaffected, it was shown in another uncontrolled study that FMD plus antibiotics could be beneficial for RA in some highly selected patients (154). More mechanistically, no significant changes in the peripheral blood DC or T cell population were observed upon standard non-surgical local therapy for PD (136), but myeloid DCs (mDCs) with a pro-inflammatory phenotype were reduced upon one week of antibiotic co-therapy (136). These changes in numbers of mDCs with an inflammatory phenotype to the levels of healthy control subjects was paralleled by lower Th17 to Treg ratios (136).

So far, we have only discussed the local dysbiotic periodontal colonization and the resulting inflammation that could be associated with RA, but a sufficiently large cross-sectional RA association study on the subgingival microbiome came to a negative result (89). Furthermore, it has to be kept in mind that the periodontal microbiome in RA could share relevant similarities with the microbiome on the palatinal tonsils, as it was at least demonstrated in healthy subjects (155). According to this finding, it might be worth to study whether a colonization of dysbiotic bacteria in the periodontal niches might be linked to a pathogenic antigen presentation on the tonsils in patients with RA.

## Intestinal Mucosa Immunity

### Intestinal Dysbiosis

One of the first large metagenome-wide association study (MGWAS) on the microbiome in fecal samples revealed significant differences between RA and control subjects with regard to the phylogenetic taxa, redox environment, transport, and metabolism of iron, sulfur, zinc, and arginine (156). Furthermore, significant associations were observed when the stool samples were compared to dental biofilm and saliva from the same individuals (156). Interestingly, MTX and an alternative herbal treatment partially restored the microbiome to a more normal respectively healthier state (156), an unexpected finding at that time, which resembles experimental evidence that an inflammatory status such as arthritis could act back to disrupt the mucosal integrity (59).

As to be expected are nutritional factors important modulators of the intestinal microbiome, which appears to also have important impact on systemic immunity. It was recently shown in this context that the treatment of mice with an alpha-glucosidase inhibitor affected the intestinal microbiome and alleviated CIA (141). Furthermore, vitamin D and its active 1,25 hydroxylated metabolite is not only a differentiation factor of monocytes and Th17 cells (157, 158), but vitamin D deficiency impairs the intestinal barrier function and affects the microbiome composition (74).

In 2017, N-acetylglucosamine-6-sulfatase (GNS) and filamin A (FLNA) were identified in RA as HLA-DR–presented peptide autoantigens for T and B cell responses (159). Both autoantigens had marked sequence homology with gut derived peptides from *Prevotella copri* (*Pc*.) and other gut commensals (159). Since then *Pc*. is in the focus by research on microbial species that could be implicated in the etiopathogenesis of RA (159–163). Subsequently, a *Pc*.-derived 27 kDa peptide was identified in association with new onset RA (160). In 2019, Alpizar-Rodriguez et al., reported that *Pc*. was enriched in the gut microbiome of asymptomatic European first-degree relatives of RA patients with immunity against citrullinated peptides, when their stool microbiome was compared to asymptomatic first degree relatives without ACPA (161). In the same year, *Pc*. as well as other Prevotella species were reported as being enriched in a gut MGWAS in Japanese RA patients (164). *Prevotellaceae* are also common in the periodontal pathology, but is to our knowledge not specifically associated with RA when present in the oral microbiota (165). While these studies appear to hint to potential microbial triggers of RA related immunity, it is remarkable to find in healthy subjects bearing RA-associated DRB1 alleles in association with the intestinal microbiome (30), suggesting that a RA-related genetic background could shape the microbiome.

### Immune System Activation in the Intestinal Mucosa

The importance of ACPA in RA led us to search in the literature for evidence of the presence of citrullinated peptides in the mucosa. Indeed, a recent proteome analysis revealed striking differences in abundance of citrullinated proteins in the colon mucosa in RA and in healthy controls (166). However, this study was performed in a small number of patients only and awaits replication. Furthermore, we do not know whether the citrullinated peptides represent known RA antigens.

Goblet cells and M cells are specialized epithelial intestinal cells, which are permissive for intestinal antigens from the gut lumen. Furthermore, CX3CR1^+^ expressing dendritic cells (DC) have the capacity of sampling commensal antigens in the small intestine via transepithelial intercellular dendrites into the gut lumen (167).

Mucosa associated lymphoid tissue (MALT) is the next line of defense against invading microbes. MALT has many anatomical similarities with secondary lymphoid organs of other locations, but a specificity of MALT is its immediate vicinity to in quantity more commensal than virulent microbial factors. Invading gut commensals are rapidly killed by macrophages, but intestinal DCs can contain small numbers of live commensals for several days (168). This process is highly relevant to selectively induce a protective IgA response. At the same time, immune responses to commensal bacteria need to be restricted to the regional lymph nodes, without potentially damaging the entire immune system (168).

DC subtypes are since a couple of years in the focus of research on intestinal MALT. Circulating DC are found throughout the entire intestine. They are located in the lamina propria of the small gut, where they accumulate in lymphoid aggregates or follicles such as Peyer patches. DC engulf microbial peptides, degrade them in their phagolysosomes into presentable components via MHC molecules to antigen specific T cell receptors (TCR) of their thymus selected T lymphocyte counterparts. DCs can be subdivided into resident plasmacytoid DCs (pDCs) and mDCs in secondary lymphoid organs, and into a migratory tissue derived DC phenotype. Notably, migratory DCs can be further subdivided by their surface molecule expression profile. In context of the local induction of regulatory T cells (iTreg), CD103^+^ α_E_ integrin expressing migratory DC have the selective ability to direct toward (iTreg) via production of retinoic acid, which is an important myeloid cell differentiation factor (169, 170). CD103^+^ cells are present throughout the intestinal mucosa, but can be differentiated according to their Integrin αM CD11b expression into CD103^+^CD11b^+^ DCs preferentially of the small intestine and CD103^+^CD11b^−^ DCs, which are enriched in the colonic mucosa, in Peyer's patches and lymphoid follicles (171). As a third intestinal mucosal DC population, CD103^−^CD11b^−^ DCs also express CX3CR1. This DC subset is resident under control of normal commensal conditions, but can switch into a migratory phenotype with the capacity of entering regional lymph nodes upon broad-spectrum antibiotic therapy (170). At least to be briefly mentioned in this review are type 3 immune-like cells (ILC3s) a fourth non-classical type of antigen presenting cells, which are important for the inflammatory response in the gut mucosa and at least present in the synovium of arthritic joints (172, 173).

Each DC subtype appears, depending on the DC to T cell ratio and other factors, to be associated with rather specific T cell responses: CD103^+^CD11b^−^ DCs appear to essentially foster the generation of a Th1 expression profile, but CD103^+^CD11b^+^ DCs preferentially lead to either Th17 or iTreg differentiation, while CD103-CD11b^+^ DCs appear to direct T cells to both, Th1 and Th17 responses (171, 174, 175). In the presence of IL-6 and TGF-β, T cells can either differentiate into pro-inflammatory or into immune stimulatory Th17 cells or into iTreg. Macrophage derived IL-23 is essential in directing CD4^+^ T cells into the inflammatory Th17 phenotype. Th17 cells express different IL-17 isoforms and IL-22, which exert important functions in APCs and in epithelial cells (176, 177). After having passed the regional lymph nodes, gut derived T cells, but also B cells and plasma cells could circulate throughout the entire body, and their evasion from the circulation will be directed by specific exit signs for their integrin homing receptors. The probably most prominent representative of these molecules is α4 β7 integrin, which directs the evasion of lymphocytes at intestinal blood vessels. IgA producing plasma cells should essentially be located at the gut mucosa, but specialized regulatory microenvironments are of major relevance for the local distribution of antibody secreting plasma cells in the gut or in the bone marrow (178, 179).

## Preliminary Conclusions

**Figure 2:**
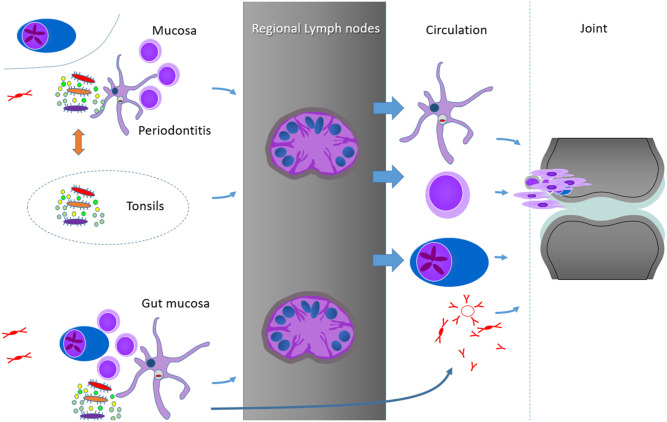
Hypothetical summary schedule of some of the possibilities to connect the mucosal sites and RA joints, from top to bottom: persistent bacterial components in DC and other phagocytosing cells, shaping of the T cell memory at mucosal sites, plasmablasts or plasma cells and soluble factors, as exemplified by antibodies (IgG monomers, IgM pentamers, and IgA dimers). Important for the control of the mucosal responses are probably the clearing regional and secondary lymph nodes. As we identified no reports on germinal center responses in the periodontal tissues, but some similarities between the subgingival periodontal and the palatinal microbiome, we include the tonsils as another possibility of mucosal activation of immune processes in RA.

We have aimed to collect as much as possible of the currently available clinical, epidemiological, and experimental evidence for the discussion of a causative link between mucosal inflammation and the emergence of RA (Figure 2). However, the amount of literature is overwhelming, and not all the potentially relevant information could be incorporated into this review simply for space and time restriction. We will now briefly discuss assembled data according to the Bradford Hill criteria of causality (13).

1. Strength of association: We identified many reports with an association, but pathogenic links between (a) periodontitis and RA, (b) oral microbiome and RA, (c) intestinal microbiome and RA as well as, (d) specific periodontal microbiota and RA were always weak.
2. Consistency: Shared epitope and the presence of ACPA were reproduced in many populations and on different continents. Reports on association of PD with RA came from different continents. Furthermore, this association was observed in populations of different ancestry. Intestinal *Prevotellaceae* were identified in association with RA in Northern America, Europe and Japan, but a Chinese study did not find this association. The microbial metagenome is probably more diverse than the human genome, but all the currently available MGWAS studies are by far smaller than the rather large and robust genetic association studies. In conclusion, although we have no formal power estimates for this statement, we believe that larger MGWAS studies are warranted.
3. Specificity: Microbes with a low level of virulence usually colonize in mixed communities. It can be assumed that this statement is likewise true for the periodontal and other localizations such as the intestinal tract. The polymicrobial synergy and dysbiosis model (PSD) is an example of how a combination of bacteria rather than a single specific species is causal in a chronic disease process like PD. Furthermore, not only *Pg*., but different periodontal pathobionts aroused suspicion to specifically trigger different important aspects of immunity in RA. Moreover, at least in experimental settings, bacteria of the oral cavity significantly affected the intestinal microbiome. In conclusion, it is from the current perspective not a single agent, but the combination of oral and intestinal microbiota as well as other potential sites of mucosal inflammation to be followed in parallel in future studies.
4. Temporality: PD diagnosis before the onset of RA perfectly fulfills this prerequisite of causality. Furthermore, a high prevalence of intestinal *Pc*. in an ACPA-positive at risk population in a cross-sectional setting appears to fulfill this criterion, but the definitive results of longitudinal studies have to be awaited, before definiteconclusions can be made.
5. Biological gradient: A dose dependency of a single causal agent, was demonstrated in several experimental settings. However, in PD, not the most severe aggressive type but the less progressive chronic form is preferentially associated with RA. Furthermore, it is currently not clear how to quantify models of polymicrobial synergy. We currently have no information of biological gradients at hands to explain the entire process of mucosal inflammation, citrullination, ACPA-specific immunity, and arthritis. Furthermore, we yet do not know how the type, the combination of synergistic factors, or the effects of time exposure for different interacting stimuli to be designed in a composite model.
6. Biological plausibility: We identified many reports about mucosal inflammation or a specific mucosal stimulus that were associated with onset or severity of experimental arthritis. Now, albeit the detailed current knowledge about the cellular mechanisms in the intestinal immune system and in periodontal inflammation is not known, it appears essential to pursue the identification of the cellular and molecular processes between mucosal inflammation and immunization in arthritis models as well as in human disease.
7. Coherence: This review is only a small extract of all the available, but to some extent contradictory knowledge, i.e., regarding specific microbial taxa being implicated in the connection of mucosal immunity and RA. Furthermore, in terms of generalizability and the transfer of data from model to disease, questions about the ACPA status, which is different in CIA and RA, as well as the different arthritis susceptibility in male and female humans and mice should be answered.
8. Experiment: Chronic PD is a hardly reversible disease, but the effects of PD therapy on RA severity are controversial. Lower incidence rates of RA in treated than in untreated PD patients go into this definition.
9. Analogy: Different periodontal pathogens cause a similar type of PD, and different oral or intestinal microbiota were associated with RA. However, it is too early to decide whether different exposures in terms of microbial taxa leading to RA can be interpreted as analog evidence for causality, or alternatively, as a violation of the criterion of specificity.

Taken together, we summarized the current viewpoints on putative mucosal triggers of RA in a narrative review. This paper is an incomplete compilation of the currently available supporting data for the postulated link between mucosal immunity and RA. In this summary of work in progress, several pieces of evidence appear to be of high validity. We conclude that ongoing major efforts, both on PD as well as the oral and intestinal microbiome, are warranted in order to answer the many remaining questions about the etiopathogenesis of RA.

## Author Contributions

AS wrote the section about periodontitis. BM wrote the other sections. All authors critically reviewed the entire manuscript for the content. In doing so, all authors agree to be accountable for the content of the work.

## Conflict of Interest

The authors declare that the research was conducted in the absence of any commercial or financial relationships that could be construed as a potential conflict of interest.
